# Complete Genome Sequence of Industrial Dairy Strain *Streptococcus thermophilus* DGCC 7710

**DOI:** 10.1128/genomeA.01587-17

**Published:** 2018-02-08

**Authors:** E. Anne Hatmaker, Lauren A. Riley, Kaela B. O’Dell, Beth Papanek, Brenton R. Graveley, Sandra C. Garrett, Yunzhou Wei, Michael P. Terns, Adam M. Guss

**Affiliations:** a Biosciences Division, Oak Ridge National Laboratory, Oak Ridge, Tennessee, USA; b Bredesen Center for Interdisciplinary Research and Graduate Education, University of Tennessee, Knoxville, Tennessee, USA; c Department of Genetics and Genome Sciences, Institute for Systems Genomics, UConn Health, Farmington, Connecticut, USA; d Department of Biochemistry and Molecular Biology, University of Georgia, Athens, Georgia, USA; e Department of Genetics, University of Georgia, Athens, Georgia, USA; f Department of Microbiology, University of Georgia, Athens, Georgia, USA

## Abstract

We report here the complete genome sequence of *Streptococcus thermophilus* DGCC 7710. *S. thermophilus* is widely used in industrial dairy production.

## GENOME ANNOUNCEMENT

*Streptococcus thermophilus* is an economically important Gram-positive bacterium often used in starter cultures for common dairy products (1). The species has undergone extensive reductive evolution, including the loss of virulence genes and genes related to carbohydrate metabolism. Losing these genes allowed for adaptation to the milk and yogurt environment, for example, through the inclusion of exopolysaccharide (EPS) and lactose fermentation operons (2). Genomic comparisons are necessary to understand such adaptive evolution and to explore other features of interest, including defense mechanisms. The *S. thermophilus* strain DGCC 7710 is of particular interest due to its role in the discovery of clustered regularly interspaced short palindromic repeat (CRISPR)-Cas systems as a bacterial immune system (3, 4), yet to date, no complete genome sequence is available.

*S. thermophilus* DGCC 7710 was grown in M17 medium with 10% lactose. High-molecular-weight genomic DNA was isolated using the Genomic-tip 100G kit (Qiagen, Valencia, CA, USA) and sequenced by the Department of Energy’s Joint Genome Institute using single-molecule real-time (SMRT) sequencing (5) on a PacBio RS II system (Pacific Biosciences, Menlo Park, CA). An additional short-read library was prepared using the TruSeq DNA PCR-free kit (Illumina, San Diego, CA, USA). The average size of the library DNA was determined using a TapeStation 2200 instrument (Agilent Technologies, Santa Clara, CA, USA). DNA concentration was measured with a Qubit 2.0 fluorometer (Invitrogen, Carlsbad, CA, USA) and adjusted according to Illumina specifications. Libraries were sequenced on an Illumina MiSeq platform with a 160-bp single-end protocol.

In total, 749,014 RS II and 2,859,266 MiSeq reads were generated for *S. thermophilus* DGCC 7710. *De novo* assembly was completed using SPAdes (6), with the Illumina MiSeq single-end reads as the short-read input and the PacBio filtered subreads input as an additional library. Scaffolds under 500 bp with low coverage were removed. The coverage of the remaining six scaffolds was over 120×. We used the complete genome of *S. thermophilus* MN-BM-A02 (GenBank accession number CP010999) as the template for genome organization. Gaps between the five larger scaffolds were filled using the final scaffold containing the ribosomal operon, which is repeated several times throughout most microbial genomes.

The complete *S. thermophilus* DGCC 7710 genome is 1,851,207 bp in length and consists of a single circular chromosome with 39% GC content. Annotation using the NCBI Prokaryotic Genome Annotation Pipeline (7) revealed 1,887 protein-coding genes and 75 RNA genes. The genome contains 56 tRNAs, 4 noncoding RNAs (ncRNAs), and 15 rRNA genes. Of the 1,887 protein-coding genes, 230 are annotated as pseudogenes, which is not unusual in *S. thermophilus* strains, such as ND07 (GenBank accession number CP016394), which has 236 pseudogenes, and ACA-DC 2 (GenBank accession number LT604076), which has 217 pseudogenes. Compared to other sequenced *S. thermophilus* strains, DGCC 7710 is of similar size and contains a comparable number of genes. Our complete assembly will allow researchers to explore the genomic diversity of *S. thermophilus* in more depth, impacting basic and applied research on a species relevant to the dairy industry.

### Accession number(s).

The complete genome sequence of *Streptococcus thermophilus* DGCC 7710 has been deposited in GenBank under accession number CP025216.
